# A Deep Residual Neural Network for Image Reconstruction in Biomedical 3D Magnetic Induction Tomography

**DOI:** 10.3390/s22207925

**Published:** 2022-10-18

**Authors:** Anna Hofmann, Martin Klein, Dirk Rueter, Andreas Sauer

**Affiliations:** 1 Institute of Natural Sciences, University of Applied Sciences Ruhr West, D-45479 Mülheim an der Ruhr, Germany; 2 Institute of Measurement Engineering and Sensor Technologies, University of Applied Sciences Ruhr West, D-45479 Mülheim an der Ruhr, Germany

**Keywords:** magnetic induction tomography, image reconstruction, inverse problems, machine learning, deep learning

## Abstract

In recent years, it has become increasingly popular to solve inverse problems of various tomography methods with deep learning techniques. Here, a deep residual neural network (ResNet) is introduced to reconstruct the conductivity distribution of a biomedical, voluminous body in magnetic induction tomography (MIT). MIT is a relatively new, contactless and noninvasive tomography method. However, the ill-conditioned inverse problem of MIT is challenging to solve, especially for voluminous bodies with conductivities in the range of biological tissue. The proposed ResNet can reconstruct up to two cuboid perturbation objects with conductivities of 0.0 and 1.0 S/m in the whole voluminous body, even in the difficult-to-detect centre. The dataset used for training and testing contained simulated signals of cuboid perturbation objects with randomised lengths and positions. Furthermore, special care went into avoiding the inverse crime while creating the dataset. The calculated metrics showed good results over the test dataset, with an average correlation coefficient of 0.87 and mean squared error of 0.001. Robustness was tested on three special test cases containing unknown shapes, conductivities and a real measurement that showed error results well within the margin of the metrics of the test dataset. This indicates that a good approximation of the inverse function in MIT for up to two perturbation objects was achieved and the inverse crime was avoided.

## 1. Introduction

In biomedicine, tomography is crucial in diagnosing various diseases. Among the noncontact methods in use are, for example, computed tomography (CT) or magnetic resonance imaging (MRI). These two methods are very effective and have a high resolution; however, they have drawbacks, such as being expensive, using dangerous radiation or being very time-consuming. An alternative tomography method is magnetic induction tomography (MIT), which has a very straightforward operating principle. Here, an alternating current flows through a transmitter coil and generates a magnetic field: the primary field. This induces an electric field in the object under examination, causing an eddy current to be excited. The distribution of the eddy currents depends on the conductivity distribution of the object. In turn, these eddy currents generate another magnetic field—the secondary magnetic field—which is finally measured by receiver coils. Various algorithms can be used to reconstruct the conductivity distribution using the receiver signals. Typically, two problems are set up for this purpose: the forward problem, which describes the physics for calculating the measured signal, and the inverse problem, which is used to reconstruct the unknown conductivity distribution from the measured signals.

MIT offers multiple advantages. The cost is very low compared to other imaging methods; it is contactless and, because of the low energy fields, it is harmless to the human body, hence being ideal for use in medical imaging. Some areas of application could be for lung diagnosis [1] or brain haemorrhages [2]. However, MIT still has some major drawbacks. Because of the dispersed nature of the electromagnetic fields, MIT has a lower resolution than, for example, MRI or CT [3]. Another problem is that almost all MIT setups so far have suffered from low sensitivity in the middle of a weakly conducting body. A recently published MIT setup investigates the origin of this low sensitivity and presents an experimentally verified solution to it that can increase the sensitivity in the middle of the weakly conducting body by more than 26 dB [4,5].

However, a further challenging problem is the solution of the inverse problem. By nature, it is ill-conditioned because only small perturbations in the signal can cause large reconstruction errors [3]. Solving this ill-conditioned inverse problem in MIT and other tomography methods has been a long-standing research topic. The typically used methods are, for example, Gauss–Newton [6], Tikhonov regularisation [7] or Landweber iteration [2]. These algorithms use a linearisation of the nonlinear original problem but usually suffer from artefacts or have a large error margin. Furthermore, iterative algorithms such as the Landweber iteration need a long computation time to approximate the sensitivity matrix [8].

In the past few years, neural networks (NNs) and other machine learning algorithms have been on the rise, to solve inverse problems in image reconstruction efficiently [9]. The nonlinear nature of NNs makes them very suitable for approximating nonlinear functions such as the inverse problem in MIT. NNs have been applied in other tomography methods such as electrical impedance tomography (EIT) [10] or MRI [11]. In MIT, there are applications of multilayer perceptrons (MLP) [12], Convolutional Neural Networks (CNNs) in combination with generative adversarial networks (GANs) [13] or Autoencoders [14].

In image reconstruction, CNNs have been extensively researched. Although originally being used for computer vision and image analysis, in recent years they have often been applied in image reconstruction. The various applications in image reconstruction include image denoising [15], super-resolution [16] or direct image reconstruction [17]. However, deep CNNs often suffer from the vanishing gradient problem [18]. For this reason, skip connections or residual neural networks (ResNets) are commonly used to avoid this issue.

Here, such a deep residual neural network is proposed for direct image reconstruction in MIT. The inverse problem was formulated as a regression task. Inputs are simulated signals based on the MIT setup introduced in [4,5]. The resulting outputs of the network are the conductivity distribution of a cuboid body with a volume of 33.6 L discretised in voxels. For training and testing, simulation data were created using cuboid perturbation objects with conductivities of 0.0 and 1.0 S/m in a background with a conductivity of 0.5 S/m. Every perturbation object had a random length between 6 and 10 cm. To avoid the inverse crime, signal creation and reconstruction were carried out on different discretisations. Furthermore, a realistic noise level of 60 dB [5] was added to the calculated signals. Once trained, the deep neural network can reconstruct the conductivity distribution of unseen data within a small error margin and even detect unknown shapes and conductivities. Finally, a real measured signal was used as test for consistency in real conditions; ResNet could reconstruct the perturbation objects within a small margin of error. This shows a good approximation of the hard-to-solve inverse problem in MIT with the presented ResNet for up to two perturbation objects.

## 2. Materials and Methods

### 2.1. The Forward Problem

#### 2.1.1. MIT Setup

In the current study, the previously published MIT setup [4,5] was used because it shows a high agreement between simulated and experimentally measured signals, which leads to a high sensitivity in the central areas because of its novel transmitter geometry. This setup has been proven successful for 3D test bodies with inhomogeneities in the centre of a weakly conductive volume. Here, this body is given as a cuboid object with the dimensions 50cm×28cm×24cm, which roughly corresponds to the dimensions of a human torso. The conductivity in a human torso varies between different types of tissue and fluid. At the chosen frequency (≈1.5 MHz), the conductivity is between 0 and 1 S/m [19]. For this reason, an average conductivity of 0.5 S/m was chosen for the background. Conductivity changes of 0 and 1 S/m were used as perturbation objects as these represent, for example, internal bleeding (≈1 S/m) or a pneumothorax (0 S/m).

**Figure 1:**
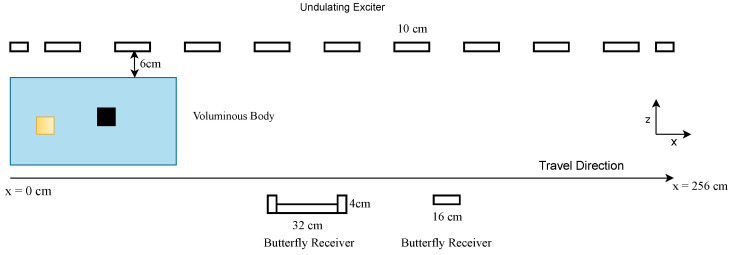
MIT setup used. A given body travels between the exciter and receiver arrays, and each cm in the x-position contributes one value to the total signal.

During a measuring procedure, the test body travels through the 256 cm-long measuring range between the transmitter and receiver array, as shown in Figure 1. The space between the body and the exciter and receiver arrays is filled with air, which means that there is a conductivity of 0 S/m between the body and the exciter and receiver.

**Figure 2:**
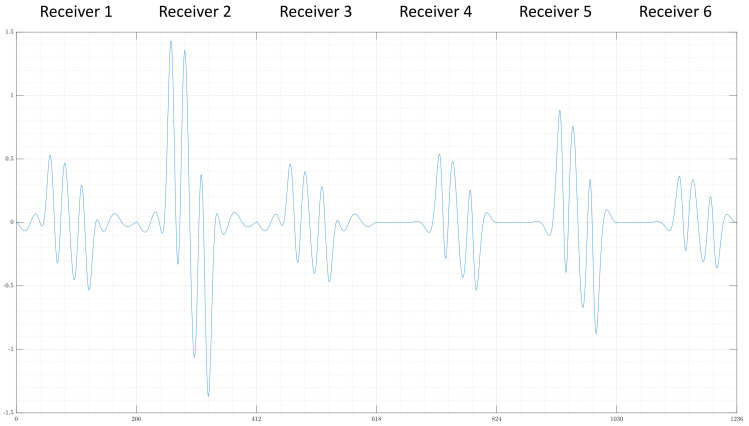
An example signal given by the forward problem, each receiver contributes 206 values to the total signal because unnecessary measuring points are cut from the original signal.

The exciter consists of 11 vertically aligned parallel wires with antiparallel current directions; this is called the undulator. Here, six receivers are used, which have a butterfly geometry and are aligned in a gradiometric position to compensate for the primary field of the undulator. In addition, the receiver array consists of three wide and three narrow receivers, each vertically stacked on top of the other. The two different geometries improve the depth resolution (z-direction), and the vertical alignment supports the resolution in the y-direction. While the object is moving through the measuring range (x-direction), the receivers record a signal for each cm passed; one example signal of all six receivers is shown in Figure 2. For simplicity and because the edge values do not give further information, the given signal for each receiver is cut so that each receiver contributes to the total signal for 206 measuring points instead of 256. For further details on the practical setup, see [5]. Here, the dataset consists purely of simulation data based on this setup.

#### 2.1.2. Theory of the Forward Problem

The underlying theory for determining the forward problem consists of Maxwell’s differential equations and the resulting eddy currents. Maxwell’s equations in time-harmonic form [20] are given by

(1) $$\begin{array}{cc} \nabla \cdot \vec{E} & =\frac{\rho }{\varepsilon }, \end{array}$$

(2) $$\begin{array}{cc} \nabla \cdot \vec{B} & =0, \end{array}$$

(3) $$\begin{array}{cc} \nabla \times \vec{E} & =-i\omega \vec{B} \end{array}$$

(4) $$\begin{array}{cc} \nabla \times \vec{B} & =\mu \varepsilon i\omega \vec{E}+\mu \vec{J}, \end{array}$$

where $\vec{E}$ denotes the electrical field, $\vec{B}$ is the magnetic flux density, ρ is the electric charge density, ε is the permittivity, μ is the permeability, *i* is the imaginary unit, ω is the angular frequency and $\vec{J}$ is the electric current density. In this context, the magnetostatic case is assumed.

Furthermore, let σ be the conductivity of a given material, then Ohm’s law [20] is given by:

(5) $$\vec{J}=\sigma \vec{E}.$$

Let $\vec{A}$ be a magnetic vector potential satisfying the equation

(6) $$\nabla \times \vec{A}={\vec{B}}_{ex},$$

where ${\vec{B}}_{ex}$ denotes the magnetic flux density of the undulator under the presupposition of weak coupling. Substituting (6) into Faraday’s law of induction, given by (3), results in the equation

(7) $$\nabla \times (\vec{E}+i\omega \vec{A})=0.$$

If the rotation of a vector field is zero, however, a scalar electric potential φ exists so that the following relationships holds:

(8) $$\vec{E}+i\omega \vec{A}=-\nabla \varphi .$$

The scalar potential φ is calculated by the method described in [21]. For this, the given body is discretised into equidistant voxels of equal size and is interpreted as an electrical network. In the middle of each voxel, the scalar potential φ is evaluated based on Ohm’s law: the resistance is calculated using the given conductivities, while the vector potential serves as a voltage source. The unknown variable φ is calculated by solving a system of linear equations, which follows from classical nodal analysis in electrical networks.

The vector potential $\vec{A}$ of the undulator and the receivers is determined analytically using Biot Savart’s law [4].

Finally, the eddy currents *J* can be calculated with the help of φ, $\vec{A}$ and (5). Using the Geselowitz theorem [22], the signal *S* is calculated by

(9) $$S=\int_{V}\vec{J}\cdot \vec{A_{R}}\;dV,$$

with *V* a given body and $A_{R}$ the vector potential of the receivers.

Because the MIT setup from [4,5] is used, there are six receivers, each contributing to the signal via 256 positions. The edge values of the signal do not give additional information; thus, the number of positions were reduced to 206. The total signal then accumulates to the following dimension:

$$S\in R^{206\cdot 6}.$$

### 2.2. The Inverse Problem

Let *F* be the nonlinear forward operator; that is, it defines a mapping

(10) $$\begin{array}{cc} F:{\left[0,1\right]}^{n} & \to R^{1236}, \end{array}$$

(11) $$\begin{array}{cc} \sigma & \mapsto S, \end{array}$$

with *n* being the number of voxels in the body. The interval [0,1] serves as the domain because the conductivities are in this range to simulate a biomedical body.

The mapping (10) and (11) is ill-conditioned. This means that only small perturbations in the signal *S* can cause large errors in the reconstruction of the conductivity distribution [2,3].

In iterative algorithms, a nonlinear least squares method for the inverse problem is set up, which is then linearised. This linearisation is usually carried out by the Jacobian matrix, which is often called the sensitivity matrix. However, calculating an approximation of the Jacobian matrix is very time-consuming because one must compute a complete forward problem once for each voxel in each iteration step.

Other problems arise from the properties of the Jacobian: usually, the condition number of the Jacobian is extremely large and, therefore, the resulting inverse problem is numerically very difficult to solve. For this reason, regularisation techniques are commonly used. However, even then, inversion is still numerically unstable.

Deep learning methods usually avoid these problems. Their ability to approximate complicated nonlinear problems makes them very suitable for approximating the inverse problem of MIT. Another advantage is the time needed for reconstruction. Once trained, they can predict the conductivity distribution of a body nearly in real time [9].

### 2.3. Dataset

The dataset used here consists of simulation data based on the real MIT setup from [4,5]. The dimensions of the body to be reconstructed are 50cm×28cm×24cm. This body is equidistantly discretised into voxels with an edge length of 1 cm resulting in a total number of 33,600 voxels. The base conductivity in each voxel is set to 0.5 S/m.

The perturbation objects in the body are of a cuboid shape, here with a randomly determined length between 6 and 10 cm in each direction. Cuboid objects proved to be practical because of the used voxel grid and for a better comparison with previous work on the used MIT setup. As shown in [5], perturbation objects with a size bigger than 0.2% of the relative body volume can be reconstructed. This is why a minimum size of 6 cm is chosen to ensure the perturbations are clearly measurable.

Up to two of those perturbation objects are randomly placed into the body with a respective conductivity of 0.0 or 1.0 S/m. These perturbation objects could simulate, for example, a pneumothorax (≈0 S/m) or internal bleeding (≈1 S/m). Because the associated signals for perturbation objects with 1.0 S/m are much weaker than those with 0.0 S/m, the signal to every possible conductivity combination of the up to two objects was calculated. Specifically, for the case with only one perturbation object, this means the signal to both possible conductivities, 0.0 and 1.0 S/m, was calculated for each randomly placed cuboid object. In the case of two perturbation objects, there are four possible conductivity combinations; that is, both objects have the same conductivity of 1.0 or 0.0 S/m, or one has a conductivity 0.0 S/m and the other has one of 1.0 S/m.

To each of the calculated signals, a realistic noise level of 60 dB was added and the corresponding differential signal was determined. This signal is the difference between a single measurement with and a single measurement without perturbation objects. These differential signals are actually very close to measurements of the real setup, as shown in [5], and are computed as follows: Let $S_{\mathrm{norm}}$ be the associated signal of the body with no perturbation objects and $S_{E}$ be the signal with perturbation objects. After adding noise of 60 dB to $S_{\mathrm{norm}}$ and $S_{E}$, the differential signal $S_{\mathrm{Diff}}$ is given by

(12) $$S_{\mathrm{Diff}}=S_{E}-S_{\mathrm{norm}}.$$

Currently, using differential signals is necessary because the total signals of simulations and real measurements differ too much from each other. On the other hand, the differential signals are actually very similar as shown in [5].

In total, 3000 cases of one perturbation object have been randomly determined, which means that the dataset contains 3000·2=6000 samples of one perturbation object because the signal to both possible conductivities is calculated.

In the case of two perturbation objects, they are randomly placed in the body, and the signals to all four possible conductivity combinations are calculated. The dataset contains signals for 15,000 cases with two perturbation objects, which means, in total, there are 15,000 · 4 = 60,000 samples. Thus, the whole dataset has 66,000 examples. Each determined signal is stored as a one-dimensional vector of dimension 1236.

**Figure 3:**
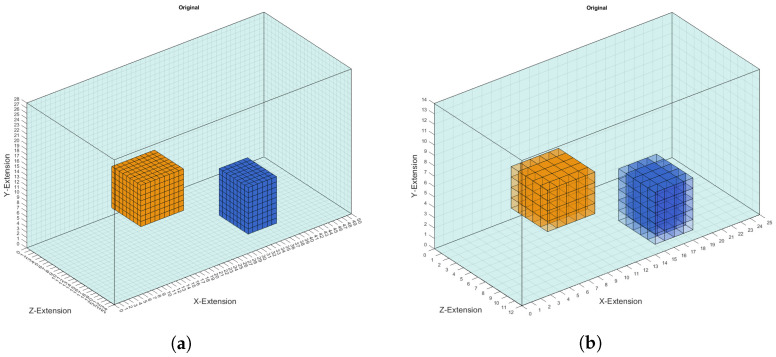
(**a**) Two cuboid perturbation objects set in the voluminous body discretised with 1 cm voxels. The yellow object has a conductivity of 1.0 S/m and the blue one a conductivity of 0.0 S/m. (**b**) The same object as in (**a**) is shown in the coarser grid of 2 cm voxels. The perturbation objects now have conductivity values differing from the original conductivity of 1.0 and 0.0 S/m on the edge of the objects.

To avoid the inverse case [23], the conductivity distribution was reconstructed on a coarser grid containing voxels of size 2 cm instead of the previous 1 cm voxel grid. More precisely, this means that, first, on the 1cm grid the perturbation objects are randomly placed; then, the corresponding signal was calculated and a realistic noise of 60 dB was added; and, finally, the reconstruction of the conductivity distribution was executed on the coarser 2 cm voxel grid. Adding noise further prevents the inverse crime from being committed. The representation of the perturbation objects on the coarser grid causes areas to exist that also contain conductivities other than 0.0, 0.5 or 1.0 S/m, as shown in Figure 3. This is because the conductivities of the coarser grid were calculated as the average conductivity of 8 voxels from the finer 1 cm grid. The conductivities of those voxels on the edges are mostly 0.25 or 0.75 S/m but can also have different values, here depending on the position of the perturbation objects. Each conductivity distribution in the coarser grid is saved as a vector of size 4200 because that is the number of voxels in that discretisation. The conductivities of the voxels are saved in a Fortran index order so that the conductivity distribution of a one-dimensional prediction can easily be transformed back into the original 3D body.

#### Data Preprocessing

Before training, standardisation is used to preprocess the data. To obtain this, the sample mean is needed, which is given by [24]

(13) $$m_{x}=\sum_{i=1}^{N}\frac{x_{i}}{N},$$

where *N* is the sample size. This can then be used to calculate the standard deviation [24]:

(14) $$s_{x}=\sqrt{\left(\frac{1}{N}\sum_{i=1}^{N}{\left(x_{i}-m_{x}\right)}^{2}\right)}.$$

With Equations (13) and (14), the standardisation of each data sample $x_{i}$ is given by the formula [24]

(15) $$z_{i}=\frac{x_{i}-m_{x}}{s_{x}},\;i=1,\ldots ,N.$$

After standardising the data, the correlation of the individual measurement points with each other is examined. The results show a high correlation between neighbouring measurement points, which means that many do not give new information for solving the inverse problem. For this reason, an extra layer to compress the signal has been implemented in ResNet.

### 2.4. Structure of ResNet

There are different ways to solve the inverse problem with machine learning methods. Here, the problem is formulated as a regression task, which means that the output layer of the used neural network consists of continuous values. In this particular case, these values lie in the interval [0,1].

For the input layer, the special geometry of the MIT setup was taken advantage of. A signal is composed of the values of six receivers each. The values of the individual receivers can be viewed one after the other, as shown in Figure 2. However, this has two major disadvantages. On the one hand, the connection between a measuring point and respective measured values of all six receivers at the same time is lost, and on the other hand, the transitions from one receiver to the next create unnatural, nondifferentiable points.

For this reason, the signals from the individual receivers were combined into a two-dimensional matrix of dimensions 6×206. Thus, each column represents all the values of the receivers to the respective measuring position.

**Figure 4:**
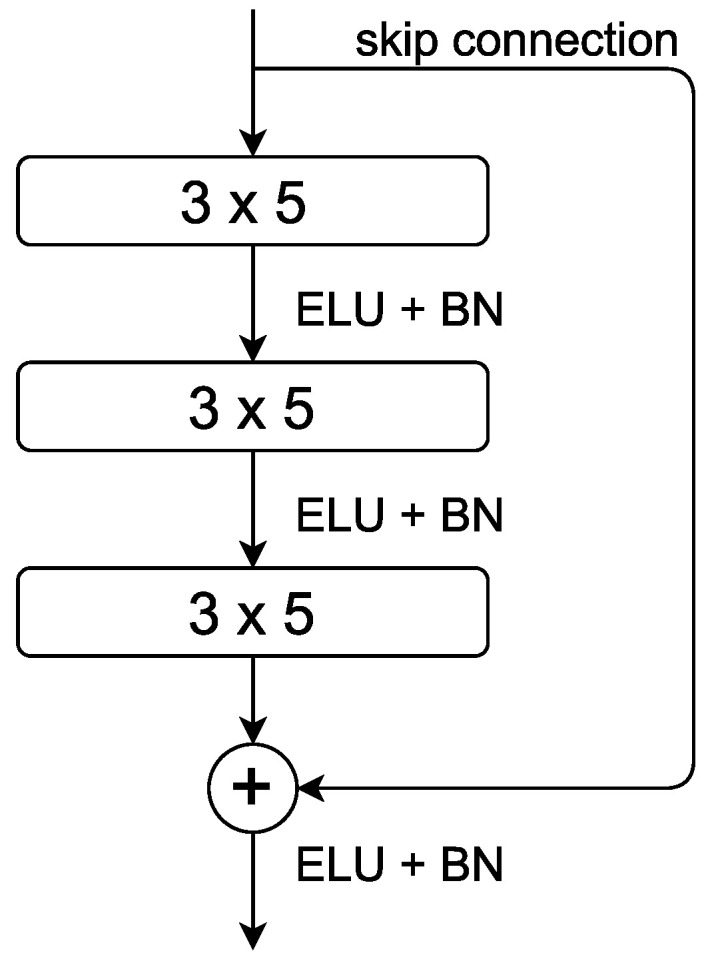
Illustration of the residual blocks used here. Each layer has a convolutional block with 3×5 filter and ELU activation. After each block, batch normalisation was used.

To exploit the receiver structure and make the network sufficiently deep, a deep residual neural network with 2D CNN layers was used. Therefore, the input layer has the dimensions (6,206,1). The base structure is similar to the one in [18], though other kernel sizes for the individual residual blocks were used. Zero padding was used to maintain the same dimensions between all convolutional and pooling layers. The residual blocks are arranged as follows: One residual block, as shown in Figure 4, consists of three consecutive 2D convolutional layers with a kernel size of 3×5. Exponential Linear Unit (ELU) activation is used after each layer, followed by batch normalisation (BN). The input size was reduced via bottleneck modules, as shown in [18]. The structure of the kernel sizes remains the same in the bottleneck layers, but one convolutional layer with a kernel size of 1×1 and strides of 1×3 to reduce the input in this block was added. Furthermore, the first convolutional layer in this module has a stride of 1×3. The strides and kernel sizes are asymmetrical to account for the assymetrical input and, in the case of the bottleneck layers, to compress the signal so it has fewer positions.

**Figure 5:**
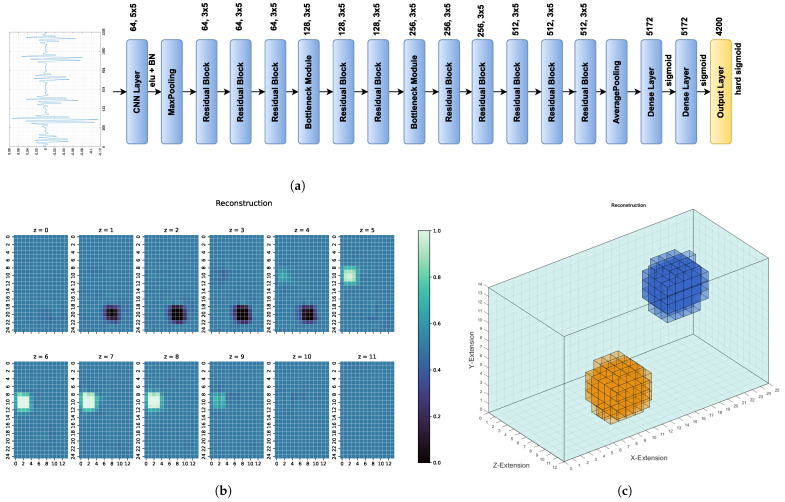
(**a**) Structure of theused ResNet. On the left is a typical differential signal, which is transformed into the 2D input and then used to reconstruct the conductivity distribution. (**b**) 2D reconstruction of the conductivity distribution by ResNet based on the differential signals, as shown in (**a**). (**c**) The same conductivity distribution reconstruction as in (**b**), only shown as 3D body.

Using the structure proposed in [18] as a guideline, the residual network was built, as shown in Figure 5a. First, with the help of one two-dimensional CNN layer and one max pooling layer, the input size was reduced because of the high correlation between the neighbouring signal points. This CNN layer has 64 kernels of size 5×5 with ELU activation and the max pooling layer a filter size of 2×2. This is followed by the residual blocks and bottleneck layers. It is important to use three layers in every residual block because a ResNet with only two layers, as proposed in [18], results in worse reconstruction results. Here, each convolutional layer follows a batch normalisation layer. After the residual blocks, one average pooling layer with a filter of size 2×2 is used. This layer is followed by two dense layers of size 5172, each with sigmoid activation. The output layer has a size of 4200 to match the number of voxels. In the output layer, hard sigmoid activation has been proven to be advantageous because the conductivities are between [0,1]. ResNet has better results with the hard sigmoid instead of the standard sigmoid activation because it is easier to achieve the output values 0 or 1 with the former one. This result has been confirmed via cross-validation.

The resulting conductivity distribution is then plotted as sections along the z-axis, as shown in Figure 5b. An example, how the two-dimensional reconstruction can be transformed back into a three-dimensional body is shown in Figure 5c.

### 2.5. Initialisation and Training of ResNet

Before training, all weights of the residual neural network are initialised with random normal weights with a mean of 0.0 and standard deviation 1.0. The whole dataset of 66,000 examples is split into 65% training, 25% test and 10% validation data. Other splits were tested, reducing the training set resulted in a decrease of the evaluated metrics, whereas increasing the training set up to 80% of the whole dataset only gave an improvement in the fourth decimal of the metrics or no improvement at all. The training set consists of 49,500 samples and is used to train the network. To avoid overfitting, regularisation techniques are commonly used. Here, the network is regularised with the help of early stopping. To achieve this, the loss of the validation dataset is monitored during training. If the loss of the validation data does not decrease for 15 epochs, training is stopped.

The loss function used here is the Huber loss function [25], which is given by the following:

(16) $$L\left(y,\hat{y}\right)=\left\{\begin{array}{cc} \frac{1}{2}{\left(y-\hat{y}\right)}^{2} & \mathrm{for}\;|y-\hat{y}|\leq \delta \\ \delta \left(|y-\hat{y}|-\frac{1}{2}\delta^{2}\right) & \mathrm{otherwise}. \end{array}\right.$$

Here, *y* is the correct output, $\hat{y}$ the predicted output of the network and δ a tuning parameter. Let *n* be a given sample size of the data, that is, $y=\{y_{1},\ldots ,y_{n}\}$ and $\hat{y}=\left\{{\hat{y}}_{1},\ldots ,{\hat{y}}_{n}\right\}$; then, the Huber loss takes the following form:

(17) $$L\left(y,\hat{y}\right)=\left\{\begin{array}{cc} \frac{1}{n}\sum_{i=1}^{n}\frac{1}{2}{\left(y_{i}-{\hat{y}}_{i}\right)}^{2} & \mathrm{for}\;|y_{i}-{\hat{y}}_{i}|\leq \delta \\ \frac{1}{n}\sum_{i=1}^{n}\delta \left(|y_{i}-{\hat{y}}_{i}|-\frac{1}{2}\delta^{2}\right) & \mathrm{otherwise}. \end{array}\right.$$

The Huber loss combines the advantages of mean squared error (MSE) and mean absolute error (MAE). The mean squared error is given by the following formula [24]:

(18) $$MSE\left(y,\hat{y}\right)=\frac{1}{n}\sum_{i=1}^{n}{\left(y_{i}-{\hat{y}}_{i}\right)}^{2}.$$

The mean absolute error is given by the following formula: [14]:

(19) $$MAE\left(y,\hat{y}\right)=\frac{1}{n}\sum_{i=1}^{n}\left|y_{i}-{\hat{y}}_{i}\right|.$$

The MSE is strongly influenced by outliers, whereas MAE weighs all errors on the same linear scale. To obtain a more robust loss function, the Huber loss behaves like the mean squared error for smaller errors and linearly like MAE if the error is bigger than the given threshold δ. Here, different values of δ between 0.05 and 0.5 have been tested via cross-validation because the conductivity values are between 0 and 1. Training and testing has shown that it is important to decrease MAE to a sufficiently small value to obtain good reconstruction results.

At first ResNet is pretrained on the same training data with δ=1.0 and a batch size of 64 for 50 epochs. Afterwards, δ is set to 0.05, and the network is trained on the same training data for 50 epochs with a batch size of 32.

The weights are updated with the Nesterov-accelerated adaptive moment estimation method (NADAM). The starting learning rate is set to 0.001 with a learning rate schedule. When the validation correlation coefficient does not decrease for seven epochs, the learning rate is reduced by a factor of 0.1 until it reaches $10^{-5}$.

## 3. Results

### 3.1. Metrics

To evaluate the performance of the deep residual neural network, different metrics were determined. In addition to the MSE and MAE, Pearson’s correlation coefficient (CC) [25] was calculated. Let $\left\{\left(y_{1},{\hat{y}}_{1}\right),\ldots ,\left(y_{n},{\hat{y}}_{n}\right)\right\}$ be *n* pairs of data; then, Pearson’s correlation coefficient is given by the following:

(20) $$r_{y,\hat{y}}=\frac{\sum_{i=1}^{n}\left(y_{i}-m_{y}\right)\left({\hat{y}}_{i}-m_{\hat{y}}\right)}{\sqrt{\sum_{i=1}^{n}{\left(y_{i}-m_{y}\right)}^{2}}\sqrt{\sum_{i=1}^{n}{\left({\hat{y}}_{i}-m_{\hat{y}}\right)}^{2}}}=\frac{s_{y\hat{y}}}{s_{y}s_{\hat{y}}},$$

where $s_{y\hat{y}}$ denotes the covariance, and $s_{y}$ as well as $s_{\hat{y}}$ the standard deviation of the data samples $y=\{y_{1},\ldots ,y_{n}\}$ and $\hat{y}=\left\{{\hat{y}}_{1},\ldots ,{\hat{y}}_{n}\right\}.$ As stated in (13), $m_{y}$ is the mean over a data sample of size *n*. All values of the CC lie between −1 and 1, where 1 indicates a positive linear relationship, −1 a strong negative relationship and 0 no relationship at all between the data pairs. That means that a CC close to 1.0 would be desirable.

Furthermore, the structural similarity index measure (SSIM) [26] has been determined, which is used for measuring the similarity between two images. The SSIM is defined as follows:

(21) $$SSIM\left(y,\hat{y}\right)=\frac{\left(2m_{y}m_{\hat{y}}+C_{1}\right)\left(2s_{y\hat{y}}+C_{2}\right)}{\left(m_{y}^{2}+m_{\hat{y}}^{2}+C_{1}\right)\left(s_{y}^{2}+s_{\hat{y}}^{2}+C_{2}\right)}.$$

The variable $C_{1}$ is calculated as the quadratic product between the dynamic range of the pixel values and a small constant $K_{1}$:

$$C_{1}={\left(K_{1}\cdot L\right)}^{2}.$$

$C_{2}$ is computed with the same formula, only with a different small constant $K_{2}$. Here, *L* is set to 1 because the conductivity lies between 0 and 1. The other parameters are $K_{1}=0.01$ and $K_{2}=0.03$. For SSIM, a value of 1 means that the reconstructed image and the original are a perfect match.

### 3.2. Results

The test dataset consists of random examples, which are separated from the total dataset before training and exclusively used for testing the network. Here, the test data consist of 16,500 samples. To ensure that the performance is not only because of randomly well-chosen test data, the test dataset was chosen to be relatively large. Furthermore, metrics on the validation set are in the same range as the test dataset.

The results for the different metrics and networks on the test dataset are shown in Table 1. For simplicity, each metric value is rounded after the fifth decimal place. Every signal of the test dataset has a noise of 60 dB added in the same way as in the training set.

**Figure 6:**
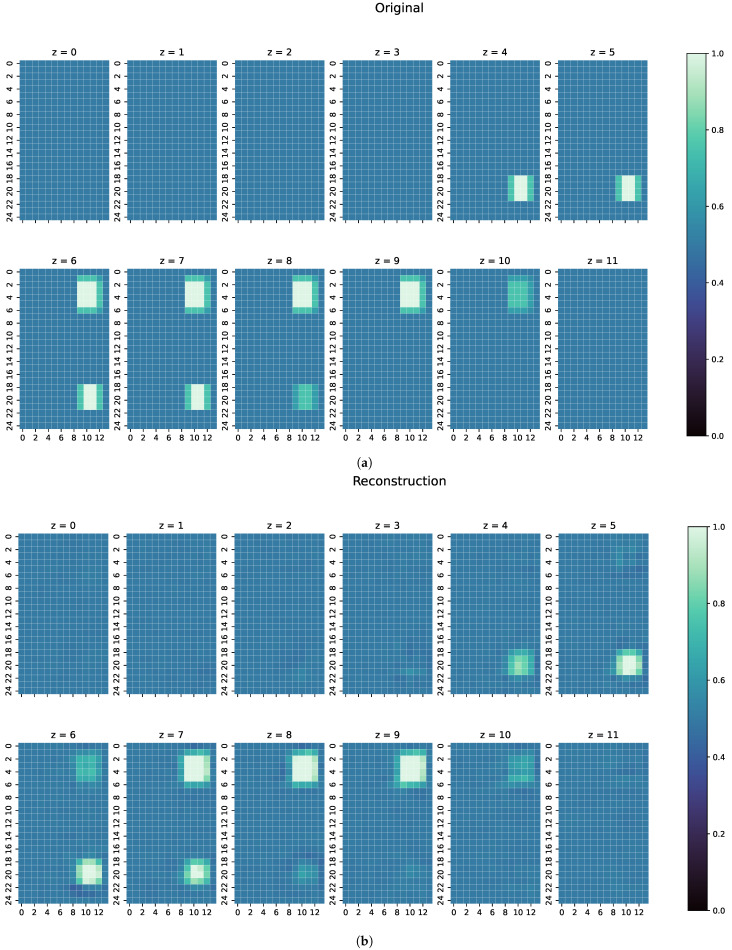
(**a**) Original conductivity distribution for one example from the test dataset. Both perturbation objects have a conductivity of 1.0 S/m, which is more difficult to detect than objects with 0.0 S/m because of the lower resulting signals. (**b**) Reconstruction of the conductivtity distribution, as shown in (**a**).

On the unseen test dataset, the proposed ResNet achieves a Huber loss value of only 0.0002, meaning there is little error. As seen before in Equation (16), the loss function is a combination of MAE and MSE, which means that the associated values are also very small: a MAE of 0.0110 and a MSE of 0.0013 is achieved. One reconstruction example can be seen in Figure 6. The metric values of that example can be seen in Table 2.

To show the generalisation ability of the network, three separate, especially challenging cases have been chosen. Because of their nature, these cases are completely unknown to the ResNet, as they contain unknown structures or conductivities:

1. The first examples consists of two overlapping cuboid perturbation objects, resulting in one big, noncuboid object. While generating the dataset, the perturbation objects have been ensured not to overlap, so that this case will be a completely unknown one for the network. The conductivity in this object has been set to 0.0 S/m.
2. The second example is one perturbation object of a conductivity not seen before. The training data only consists of objects with conductivity 1.0 or 0.0 S/m, and only on the edges of those perturbation objects do the conductivities differ from those because of the discretisation. The test case here has a conductivity of 0.8 S/m.
3. Finally, a real measurement from the MIT-Setup is tested. In the selected measurement, the difference signals between simulation and measurement differ in amplitudes, but have a similar pattern. This shows that even the reconstruction of real measurements is possible if the pattern of the signal largely corresponds between reality and simulation.

**Figure 7:**
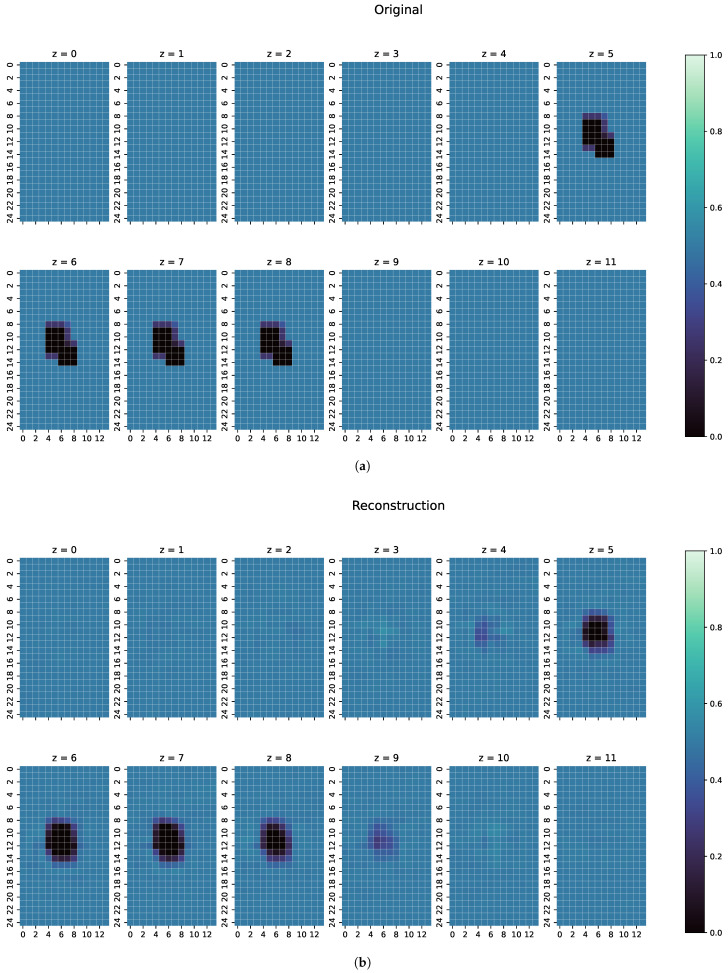
(**a**) Original conductivity distribution for case 1 with two objects overlapping each other in the centre of the body. (**b**) Reconstruction of the conductivity distribution of case 1, as shown in (**a**).

**Figure 8:**
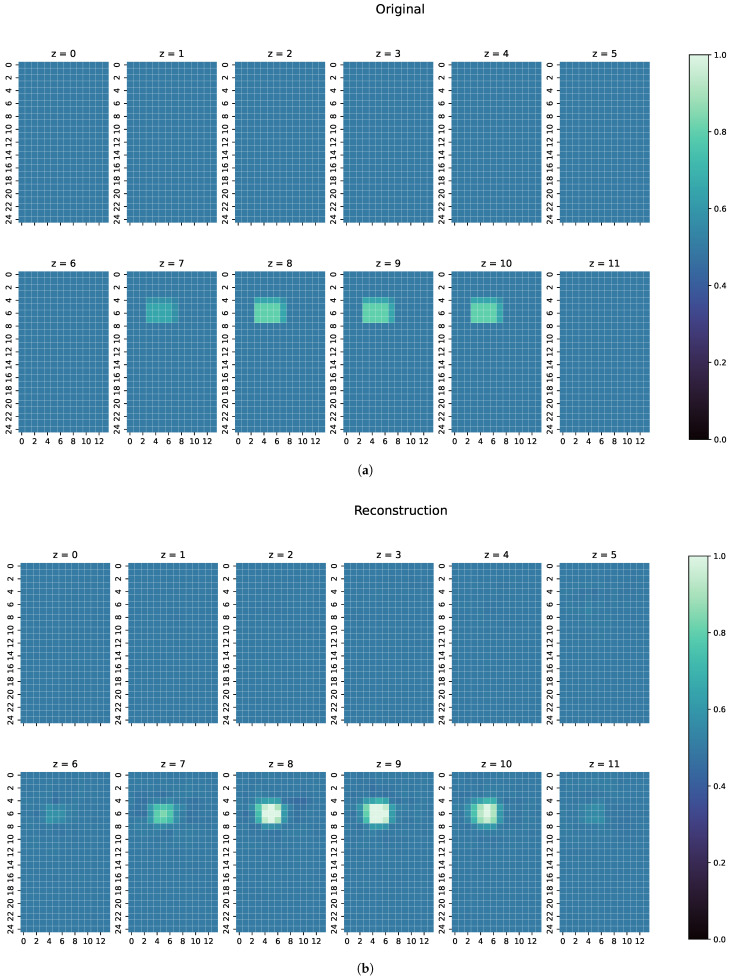
(**a**) Original conductivity distribution for case 2 with one object of a different conductivity than what has been trained. (**b**) Reconstruction of the conductivity distribution of case 2, as shown in (**a**).

**Figure 9:**
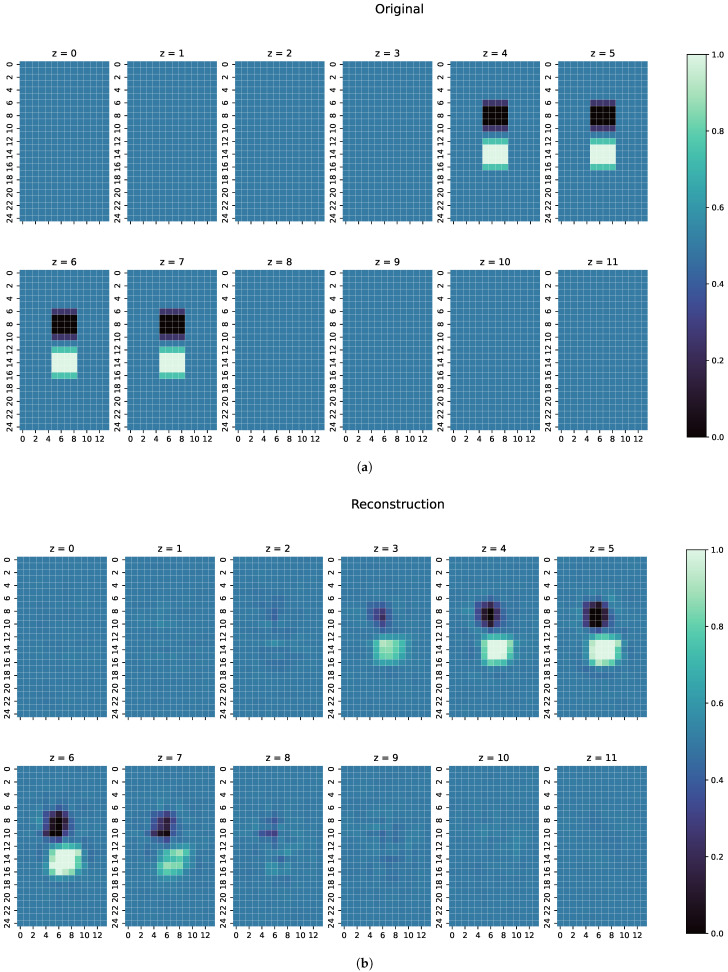
(**a**) Original conductivity distribution for case 3 of a real measurement. The body is discretised into 2 cm voxels, as in the simulation cases; thus, there are edge values with different conductivities. (**b**) Reconstruction of the conductivity distribution of case 3, as shown in (**a**).

For every one of those combinations the MAE, MSE, CC and SSIM have been computed. The results are shown in Figure 7, Figure 8 and Figure 9. An overview of all metric values for examples 1–3 can be seen in Table 2.

## 4. Discussion

An analysis of the reconstruction results of the test dataset shows that ResNet performs well on unseen data, thus producing the low values for MAE and MSE and high values for CC and SSIM, as can be seen in Table 1. The perturbation objects are reconstructed with correct conductivity and are shaped well within the range of the original measurements. The slightly blurred representation follows from the properties of the measured electromagnetic fields and indicates that ResNet is a good approximation of the real, continuous inverse function in MIT. Furthermore, as can be seen in Figure 6, perturbation objects with a conductivity of 1.0 S/m are reconstructed, despite the lower signal response compared with the other cases with a conductivitiy of 0.0 S/m. The good metrics of over 16,500 samples indicate that the position of the perturbation objects does not matter for determining the conductivity distribution in the voluminous 3D body. Thus, reconstructions of perturbation objects in the entire body are possible without any restrictions, even in the centre, which are usually difficult to detect.

In the examples intended to show the robustness of ResNet (Figure 7, Figure 8 and Figure 9), the metrics of MAE, MSE, CC and SSIM (Table 2) are in the range of the overall metrics of the test dataset (Table 1). This again confirms the assumption that the ResNet is very good at reconstructioning unseen examples and that there is little overfitting.

An individual evaluation of the examples shown in Figure 7, Figure 8 and Figure 9 was also conducted. In the first example, as shown in Figure 7, ResNet can detect the perturbation with correct conductivity and can even vaguely reconstruct the original shape. The original perturbation object is larger than the perturbation objects occurring in the training data, where only cuboid objects with a maximal length of 10 cm are included. Here, the original perturbation object has a maximum length of 13 cm. In z =6 and z =7 of the reconstruction, this length is also detected from ResNet. Some uncertainty in the reconstruction can be seen because there are small artefacts in the z-direction and unsharp edges in all directions. However, the position of the perturbation object in the reconstruction is mostly on the correct coordinates with correct conductivity.

For the second example (Figure 8), ResNet can reconstruct the perturbation object pretty close in shape and position, here with a slight uncertainty in the z-direction; most of the conductivities in the reconstructed voxels are close to the ground truth, especially on the edges of the perturbation object. However, because ResNet was only introduced to objects of conductivities 1.0 and 0.0 S/m during training, most of the middle values of the perturbation object are reconstructed close to 1.0 S/m. Nonetheless, this shows that the ResNet can detect unknown conductivities and can even approximately reconstruct the conductivity distribution with few artefacts in the entire body.

**Figure 10:**
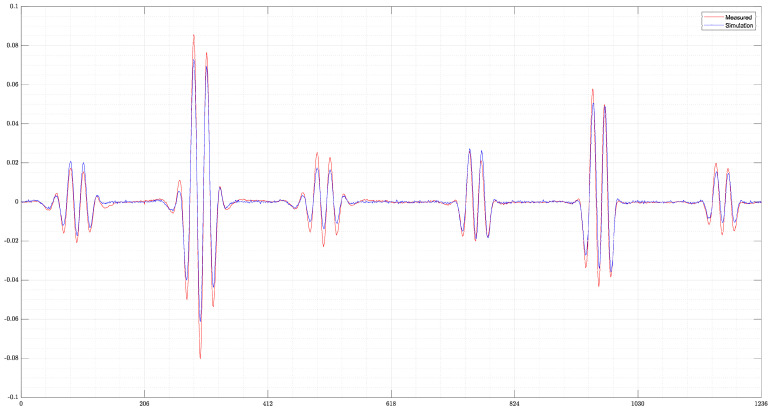
Comparison of the measured and simulated differential signals. The measured differential signal of the real MIT setup shows higher amplitudes than the simulated signal. However, the pattern of the signal is very close to the simulated one.

The final example (Figure 9) shows the reconstruction of a measured signal from the real MIT setup. A comparison of the signals can be seen in Figure 10. Despite the far higher and slightly displaced amplitudes of the real measurement, ResNet can detect the two original objects. For the perturbation object with 1.0 S/m, the position is mostly correct and the conductivities very close to the ground truth. However, the object with 0.0 S/m conductivity is slightly misplaced and smaller than in the original. Both perturbation objects are clearly separated. Especially in z-direction, the edges of the objects are unsharp, showing some uncertainty of the reconstruction at the edges of the object. Nevertheless, this shows that reconstructions of real measurements are possible. However, this can only be achieved if the real measured and simulated signals have a high degree of similarity. For measurements with stronger deviations from the calculated simulations, only reconstructions of the 0.0 S/m perturbation objects could be obtained, and the more difficult-to-detect 1.0 S/m perturbation object is then not detected by ResNet.

Overall, the results show that ResNet is not only able to reconstruct random cuboid shapes on the test dataset, but it can also detect perturbation objects of unseen shapes or conductivities. For a given close similarity between reality and simulation, also real measurements can be used for reconstruction. The metrics for all three test cases are in the error range of the results of the test dataset. Furthermore, the low metric values for the whole test dataset show that the reconstruction of perturbation objects in the whole voluminous 3D body is possible. This is especially true for those objects in the centre of the body, which are hard to detect. Because of the care in creating the dataset with noise of 60 dB and different discretisation models for the forward and inverse problem, the inverse crime has been avoided; thus, even the reconstruction from a real measurement is possible and well within the error range of the simulation reconstructions. From this, it can be concluded that a good approximation of the inverse function of MIT has been achieved, at least limited to up to two perturbation objects.

## 5. Conclusions

The current work has shown that, with given simulated data and added realistic noise, the reconstruction of the conductivity distribution in a voluminous body with up to two perturbation objects with conductivities of 1.0 and 0.0 S/m is possible. In particular, a conductivity of 1.0 S/m, which is difficult to reconstruct, is reliably detected by the presented ResNet structure on the test dataset. The test cases have shown that even unknown shapes, conductivities and real measurements are possible to reconstruct with good positional accuracy, even if the shape of the perturbation objects are still blurred.

Despite the good results, there is still room for improvement. To use the ResNet, the simulated and measured signals must be almost identical. Currently, this is not always the case. This leads to reconstructions where ResNet can only detect the perturbation objects with the easier-to-detect conductivity of 0.0 S/m and where a perturbation object with 1.0 S/m conductivity is not found. This means that an optimisation of the physical model is needed. To improve signal quality, an optimisation of the MIT setup would also be desirable.

For real-life applications on a human body, the forward model needs to be adjusted to simulate bodies closer in shape than the current cuboid body. Furthermore, a NN has to be tuned accordingly to work for only small changes in conductivity to correctly restore other conductivity distributions. The optimal would be a reconstruction that can distinguish between objects only 0.1 S/m differing from each other. 

## Figures and Tables

**Table 1:** Different metrics for ResNet on the test dataset.

Network	Loss	MAE	MSE	CC	SSIM
ResNet	0.0002	0.0110	0.0013	0.8765	0.8417

**Table 2:** Comparison of MAE, MSE, CC and SSIM for the example in Figure 6 from the test dataset and for all three given examples shown in Figure 7, Figure 8 and Figure 9.

Example	MAE	MSE	CC	SSIM
Test case	0.0092	0.0009	0.9203	0.8717
Special case 1	0.0082	0.0008	0.9104	0.8820
Special case 2	0.0114	0.0027	0.9396	0.8729
Special case 3	0.0145	0.0025	0.7892	0.7802

## Data Availability

Not applicable.
